# Content, Usability, and Utilization of Plain Language in Breast Cancer Mobile Phone Apps: A Systematic Analysis

**DOI:** 10.2196/mhealth.7073

**Published:** 2017-03-13

**Authors:** Tamar Ginossar, Sayyed Fawad Ali Shah, Andrew J West, Joshua M Bentley, Charlene A Caburnay, Matthew W Kreuter, Anita Y Kinney

**Affiliations:** ^1^ Department of Communication and Journalism University of New Mexico Albuquerque, NM United States; ^2^ Department of Strategic Communication Texas Christian University Fort Worth, TX United States; ^3^ Health Communication Research Laboratory Brown School of Social Work Washington University in Saint Louis Saint Louis, MO United States; ^4^ Department of Internal Medicine Health Science Center University of New Mexico Albuquerque, NM United States

**Keywords:** mobile phones, mobile apps, breast cancer, cancer-related content

## Abstract

**Background:**

Breast cancer is one of the leading contributors to preventable illness and death among women. Although mobile phone apps provide unprecedented opportunity to engage women along the cancer continuum, little is known about the availability, content, and usability of breast cancer mobile phone apps.

**Objective:**

This study analyzed the content and adherence to literate design standards of all breast cancer-related apps available on the App Store and Google Play, as well as the relationship between their content, user ratings, and price.

**Methods:**

Following identification and downloading of all available breast cancer mobile phone apps in October 2015, 101 apps were confirmed as focusing on breast cancer. Based on prior research, we adapted and applied a content analysis scheme that was specific to breast cancer apps, including their main purpose, relevance to the cancer care continuum, and adherence to usability standards outlined by the Institute of Medicine (IOM).

**Results:**

The most common aim of apps was educational (73/101, 72.3%), followed by behavior change (24/101, 23.9%), fundraising (20/101, 19.8%), and advocacy (14/101, 13.9%). On the cancer continuum, primary prevention (strategies to prevent cancer cells from occurring) was mentioned in almost one-third of the apps (30/101, 29.7%). Less than half of the apps (46/101, 45.5%) presented information about mammography and/or breast clinical exam, and 53 apps (52.5%) discussed breast self-exam (which is no longer recommended). Symptoms of cancer prediagnosis, such as a lump, were discussed in almost half of the apps (48/101, 47.5%) and a similar number of apps included information about genetic risk for breast cancer (47/101, 46.5%). Information about breast cancer diagnosis was included in 42 apps (41.58%) and 43 (42.6%) apps discussed treatment options. Survivorship issues were addressed in 17 (16.8%) apps. Only one (1.0%) app discussed hospice. Adherence to usability recommendations was low. The median composite score was 3 (mean 2.60, SD 1.20) of the six recommended usability items. With eight plain language items, the median of the composite health literacy score was 5 (mean 5.06, SD 2.00). Most apps did not use easy-to-understand words (44/101, 43.6%) and few (24/101, 23.8%) defined key terms.

**Conclusions:**

Current breast cancer apps provide important information about breast cancer, but the most common topic covered is breast self-examination, a non-evidence-based screening strategy. Apps that focus on evidence-based strategies on the cancer continuum are needed, with a notable pressing need for apps that would address survivorship and end of life. Finally, developers of breast cancer apps should adhere to IOM standards to meet the needs of diverse populations and reduce current disparities.

## Introduction

### Overview, Rationale, and Goals

More than 1,677,000 women worldwide are diagnosed with breast cancer and more than 522,000 die of it annually [1], making it the most commonly occurring cancer and the principal cause of death from cancer among women globally [2]. Breast cancer constitutes a major contributor to preventable cancer burden [3,4], which refers to the morbidity and mortality that can be reduced by health behavior change, access, and utilization of screening and treatment services. Notably, most of the inequity in cancer health outcomes, including international and interethnic differences in breast cancer incidence and mortality, are attributed to preventable causes [5,6]. Although evidence-based comprehensive programs have documented successes in increasing breast cancer survival rates in high resources settings, much work is needed in increasing their reach, particularly among medically underserved populations in the United States and beyond [7].

Mobile health technology, or mHealth, holds great potential in reducing disparities in cancer-related health outcomes. With nine of 10 Americans owning at least one cell phone, and a majority (63%) of these devices providing access to mobile Internet service [7], it has tremendous penetration. Unlike previous communication technologies [8], these devices are disproportionally used by members of low-resources communities, with minority users more likely to access the Internet exclusively from their mobile phone [9]. Further, well-designed mHealth holds great potential in promoting health in low-resources communities in developing [10] and developed countries [11]. In view of the international and national burden of breast cancer and the potential of mHealth in improving medical and public health practices [12], it is important to understand the use of breast cancer-related apps across the cancer care continuum. However, although half of cell phone users reported downloading apps in 2013 [13], little is known about these apps’ design, availability, and health-related use in general and in the context of breast cancer in particular. To date, studies have examined content of cancer-related apps using content analysis of apps’ descriptions on the App Store [14], of cancer-related apps available on iPhone only [15], and of studies reporting on educational cancer apps [16], yet the specific content of breast cancer-related apps available to consumers along the cancer care continuum [17] has not been examined. Studies of mHealth reveal that although apps offer beneficial functions [18], consumers are faced with a “bewildering array” of available health apps [19], with varied and often dubious quality [20,21]. Of apps that focus on cancer awareness, few discuss evidence-based preventive health behaviors [14], a deficiency that is likely because of a lack of medical professional involvement in design of apps [22]. Furthermore, cancer-related apps can pose a danger; for instance, skin cancer-related apps accurately assessed melanoma only 10% of the time [23].

To reach individuals from low-resources communities who are disproportionally affected by preventable cancer burden, literate principles should be followed in the design of apps. The Institute of Medicine (IOM) published guidelines on mHealth-literate design strategies, including plain language and appropriate usability features [24]. However, it is unknown to what degree these standards are implemented in the design of breast cancer-related apps [24].

In view of the importance of breast cancer as a public health concern, the goal of this study was to systematically analyze the availability and content of breast cancer apps available on the main platforms of Google Play and the App Store, and their main features, including content on the cancer care continuum, goals, adherence to the IOM literacy guidelines, price, and user ratings. The following sections review past research on these factors as they relate to breast cancer mHealth.

### Prevention

A growing body of evidence documents mHealth interventions’ effectiveness in engaging users in cancer preventive measures [25]. For instance, text message reminders to female patients before scheduled breast cancer screenings were found to greatly increase screening attendance [26]. Apps have also been shown to successfully increase breast cancer screenings in rural areas as well as disseminate important breast cancer information where cultural and social constraints prevent the spread of accurate breast health information [27,28].

### Treatment

Apps are also utilized to enhance care delivery during cancer treatment. These apps work as information management tools where patients can check appointments, journal symptoms, and log medications [29]. These apps and devices can also reduce the communication gap between patients and providers, expedite treatment, allow patients to more easily report side effects of chemotherapy and other treatments to health care providers in a timely manner [10,30], provide information about postchemotherapy side effect management, and assist cancer patients with medication adherence [11]. Although mHealth has great potential for cancer supportive care during treatment (eg, management of symptoms), research has not explored its role in improving patient quality of life [23,31].

### Survivorship

Survivorship is another important area for mHealth cancer care interventions. In addition to cancer recurrence, breast cancer survivors are at a greater risk of comorbid conditions, such as obesity, osteoporosis, cardiovascular disease, and diabetes [32], and many breast cancer survivors do not meet healthy lifestyle recommendations [32]. Several mHealth interventions on nutrition quality, physical activity, and improved eating self-efficacy have addressed this problem and were found to facilitate significant short-term weight loss, decrease waist circumference, and increase self-efficacy in breast cancer survivors [33]. It has been suggested that lifestyle interventions for cancer survivors, including breast cancer survivors, should incorporate text messages and mobile phone apps to augment existing survivorship interventions [34]. In addition, researchers noted the potential of apps to increase physical activity for breast cancer survivors [35] and the successful reduction of stress among breast cancer survivors in a technology-based self-management intervention [36] further demonstrates the promise of technology-based survivorship interventions.

### mHealth Literacy

User skills are key to effective utilization of mHealth, particularly among underserved communities. Digital health literacy is related to one’s ability to seek, locate, comprehend, and assess health information from electronic sources [37]. The concept of digital literacy draws on health literacy, defined as the ability to understand and use health information, communicate needs to health providers, and understand information from health care institutions [38]. Health literacy is strongly related to health outcomes, including cancer communication [39]. Similarly, digital health literacy is related to age and education, as well as to better outcomes as a result of health information seeking [40]. Use of mobile technology is dependent on both health and digital literacy skills. Digital literacy is crucial to engage in health maintenance, change behavior, and utilize health care services [41]. People with low literacy are less likely to access the Internet to seek information about health concerns [20], despite strong information needs following cancer diagnosis [42,43]. Patients with low health literacy are less likely to use health apps or perceive them as easy or useful, and hence are less likely to benefit from this technology [31]. Understanding different literacy levels is consistent with research by Second-Level Digital Divide [36] that examined different skill levels of using digital communication technologies. Health-literate apps can bridge the digital divide by improving quality and usefulness of health information and interventions that would ultimately lead to better health outcomes [24].

The price of apps is an additional factor in apps’ dissemination and adoption of health promotion messages. Paid diabetes apps demonstrated better adherence to IOM standards compared to free apps [44]. Such advantage of paid apps has the potential to limit the dissemination of health information and to increase health disparities because most users are reluctant to pay for apps [45]. However, it is unknown whether these differences are manifested in apps in other health-related content areas, including breast cancer.

Finally, although systematic reviews aim at addressing availability and analyzing the content and features of the analyzed apps use [46], mHealth allows some insight into users’ experiences by featuring user ratings of apps. User ratings have been shown to be correlated with professional quality ratings of apps [47]; therefore, exploring their association with adherence to literate app design has the potential to shed light on the relationship between design and user experience.

In view of the preceding research, the goal of this study is to evaluate the availability and content of existing breast cancer-related apps. In particular, we assessed apps’ content for intended purpose, consistency with the breast cancer care continuum, adherence to IOM plain language and usability standards, and the association between adherence to standards and apps’ prices and users’ reviews.

## Methods

### Sampling

**Figure 1:**
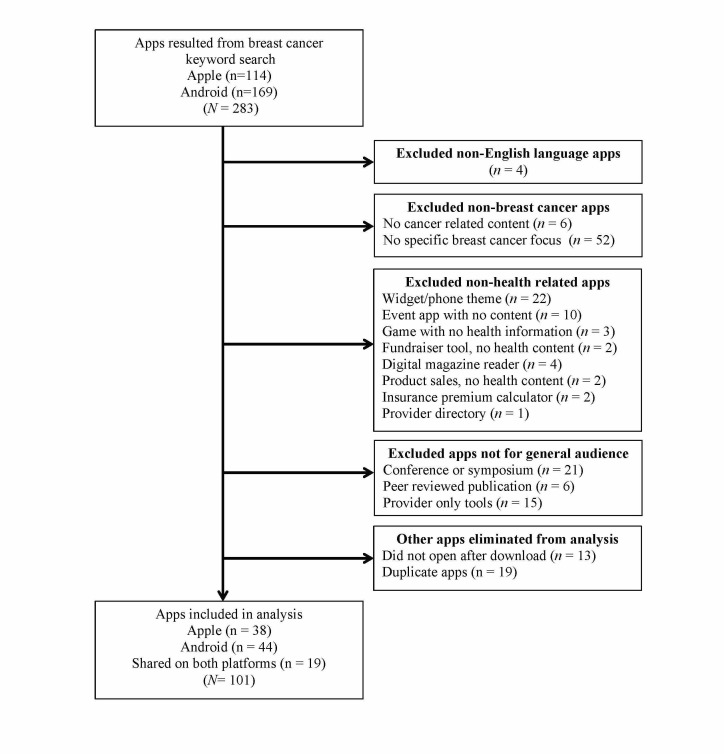
App Exclusion Chart.

The study did not involve recruitment of human population; therefore, ethics committee approval was not required. Following previous content analyses of mHealth apps [44,48], a list of breast cancer apps was generated in October 2015. Apple apps were searched directly from the App Store using an iPad device. Android apps were searched on Google Play Android App Store using an Android tablet device. The “any price” (Apple App Store) and “all price” (Google Play Android App Store) search options were selected to include both free and paid (fee-based) apps in the search results. The search term “breast cancer” on both platforms resulted in 264 unique apps (105 Apple apps, 131 Android apps) and 28 apps that were available on both platforms. Apps were chosen for analysis if they met the following inclusion criteria: (1) English-language app, (2) focused on breast cancer, (3) health related (as opposed to apps that included entertainment only, such as ringtones), and (4) intended for a general audience of consumers rather than health care professionals. A total of 163 apps were excluded (see Figure 1 for information on reasons for exclusion). One app had both a free and an upgraded paid version and the content was found to be different, so both versions were coded. The final sample of apps that were downloaded and coded in this analysis was 101, including 44 unique Android apps, 38 unique Apple apps, and 19 apps that were available on both platforms. Most of the apps were free (85/101, 84.1%) and only a minority (16/101, 14.8%) were paid apps. See Figure 1 for the process of inclusion and exclusions of apps.

### Coding Process

At the time of the study, only one prior study explored content of apps and adherence to IOM standards in diabetes-related apps [44]. Consequently, no coding scheme was available for use in the context of breast cancer. This previous IOM-related coding scheme [44] was applied, and its content-related scheme was adapted to the breast cancer context based on past literature and in particular the focus of this study on the cancer continuum. TG created the initial adaptation based on the literature and her experience as a cancer communication researcher, and three additional authors provided input based on their knowledge and experience in cancer communication, including clinical experience of the last author. Following discussions between the researchers, the coding scheme was finalized (see Table 1 for the coding scheme). Two trained graduate research assistants downloaded each app to an iPad or an Android tablet. After reviewing the app’s features, they entered information into an electronic database. The coding process began with coding general characteristics listed in the App Store and Google Play. Then, coders coded cancer-related content and adherence to the IOM recommendations (see Table 1 for a list of variables) for designing health-literate mobile apps. The coders were trained by TG for two sessions lasting a total of 5 hours and then they coded the apps individually. Two independent coders coded a sample of 30 apps (29.7% of the total sample) to test intercoder reliability. Any coding disagreements were discussed collectively with the first author until agreement was reached. For each variable, intercoder reliability was assessed using Krippondorf alpha (see Table 1).

**Table 1:** Variable categories, names, definitions, and intercoder reliabilities.

Category and name			Definition		Intercoder reliability, Krippendorf α^a^
**Purpose**					
	Information/education		Content to inform/educate		1.0	
	Behavior change/maintenance		Content to motivate, encourage behavior change		1.0	
	Fundraising		Raising money, donations		0.88	
	Advocacy		Other than fundraising		0.86	
**Cancer continuum primary prevention**					
	Primary prevention		Health promotion activities, diet, and exercise		1.0	
**Genetic risk/screening**					
	Risk		Genetic risk (eg, BRCA discussed)		0.89	
	Screening		Genetic screening discussed		1.0	
**Screening evidence-based**					
	Mammography		Mammography discussed		0.90	
**Non-evidence-based clinical breast exam**			Clinical breast exam discussed		1.0
	Breast self-exam		Breast self-exam discussed		1.0	
Symptoms prediagnosis			Cancer symptoms prediagnosis discussed/explained		0.90
**Diagnosis**					
	Stage		Cancer stages discussed		0.89	
	Tumor type		Types of tumors discussed		0.90	
	Prognosis		Prognosis discussed, including survival		1.0	
**Treatment**					
	Treatment options		Breast cancer treatment discussed		1.0	
	Side effects/symptoms		Treatment side effects discussed		1.0	
	Medication care management		Information on medication types/brands		1.0	
**Chemotherapy prevention**			Chemotherapy prevention medication		1.0
Survivorship			Life after cancer discussed		N/A
End of life			End-of-life/hospice information		N/A
**Breast cancer care**					
	Breast cancer continuum care		Breast cancer continuum care and or behaviors discussed		0.89	
Research/Science					
Biological process			Information on biological process of breast cancer		1.0
Trial recruitment			Clinical trial recruitment		N/A
Research referenced			App cites medical research studies		0.74
**Adherence to literate principles design plain language**					
	Common, everyday words		Common plain language used		1.0	
	Personal pronouns		Personal pronouns such as “you” used		0.90	
	Defined terms		Terms explained/defined		1.0	
	Active voice		Use of active voice		1.0	
	Action words		Direct action language used		1.0	
	Present tense		Present tense used		1.0	
	Short sentences		Sentences 15-20 words max		1.0	
	Limited paragraph size		Short paragraphs, use of bullets/lists		1.0	
**Usability**					
	Images for learning		Use images that facilitate learning		1.0	
	Bold colors/background		Use bold colors with contrast; avoid dark backgrounds		0.71	
	Home/menu page		Enables easy access to home/menu page		0.71	
	Back button		Back button identified as arrow or labeled		0.85	
	Simple search		Utilizes simple search tool		0.71	
	Browsing		Easy browsing/navigating through app		0.75	
**Technology**					
	Email		Connected with device email option/in-app email options		0.71	
	Calendar		Connected with device calendar		0.53	
	Reminders/notifications		Offered device notifications		0.78	
	Maps/GPS		Offered maps/GPS options		0.87	
	Print		Included print options		0.65	
	Save options		Save content as .doc, PDF, image files		N/A	
**Interactivity**					
	Personal information		Contact information input		1.0	
	Personal statistics		Input of height, weight, etc		1.0	
	Expert interaction		Interactions with medical professionals		1.0	
	Peer support		Interaction with other app users		N/A	
	Connect with event		Link user with event source		1.0	
	Audio/video features		Use of sound bite/video content		1.0	
	New media		Use of social media and/or text		0.90	

^a^ N/A: Krippendorff alpha could not be calculated due to lack of variance.

### Coding Scheme

#### General Characteristics

Basic information was captured from the App Store and Google Play, such as the provider or seller, price (if any), age rating, app category, and numbers for both ratings and reviews for each coded app and its price.

#### Purpose of the App

For perceived purpose of the app content, coders noted one or more of the following four categories: (1) information/education (eg, reference/glossary of breast cancer terms), (2) behavior change/maintenance (eg, becoming more physically active, participating in screening), (3) fundraising, and (4) advocacy (eg, awareness-raising campaigns).

#### Cancer Continuum-Related Content

To examine the apps’ foci on the cancer care continuum, one or more of the following variables were coded: (1) primary prevention; (2) evidence-based cancer screening (mammography, clinical breast exam); (3) diagnosis, including information about cancer staging, type of tumor, and information about prognosis, such as survival rate; (4) disease management/therapeutics, including information about treatment, side effects of treatments, and treatment medications and chemo prevention to prevent recurrence; (5) survivorship; and (6) end-of-life care. In addition, the following prediagnosis categories were coded: (1) genetic risk (eg, family history of cancer) and (2) breast self-exam and symptoms of breast cancer prediagnosis. Finally, the coding scheme included research and scientific-related content, which was comprised of information on the biological process of cancer, references to research, and discussions of clinical trials.

### Adherence to IOM Literate Design Principles

To assess the apps’ adherence to the IOM mHealth literacy guidelines [24], we used the coding scheme developed and tested by Caburnay and colleagues [44]. Plain language variables included the presence or absence of the following: (1) common everyday words, (2) the pronoun “you” (second person voice), (3) use of present tense, (4) defined technical terms, (5) use of active voice, (6) use of action words, (7) use of short sentences (15-20 words), and (8) limited paragraph length (including bullet points and short lists). Each of these eight variables was coded (0=not present; 1=present), and the results were summed to create a composite plain language score.

Usability was measured as a composite of (1) avoidance of dark backgrounds, (2) easy access to home page (eg, home/menu button), (3) clearly labeled back button, (4) in-app simple search, (5) enabled browsing, and (6) use of images that facilitate learning (eg, diagrams of breast anatomy). Each of these six variables was coded (0=not present; 1=present) and summed to create a composite usability score [24,44].

Variables on graphics and technology use were also recorded and were composed of integration with other device apps (email, calendar, maps, reminders, GPS) and save/print options. Interactivity variables included user-tailored/interactive content (eg, input contact information, measures such as weight and height, expert interactions, online peer support, connect user with event), use of audio and video features, and use of new media or texting (eg, Facebook, Twitter).

### Statistical Analysis

We used SPSS version 23 (IBM Corp, Armonk, NY, USA) to calculate descriptive statistics; *t* tests to identify associations between app characteristics, price (free vs paid), and user ratings; and Pearson correlations to examine the relationship between IOM guidelines and user ratings. Significance was determined at a level of alpha=.05.

## Results

### Road Map

Our goal in this study was to better understand availability and content of breast cancer-related apps available to the public, with a focus on their purpose, cancer continuum-related content, adherence to IOM literate design standards, price, and user ratings.

### Sample Description and General Characteristics

**Figure 2:**
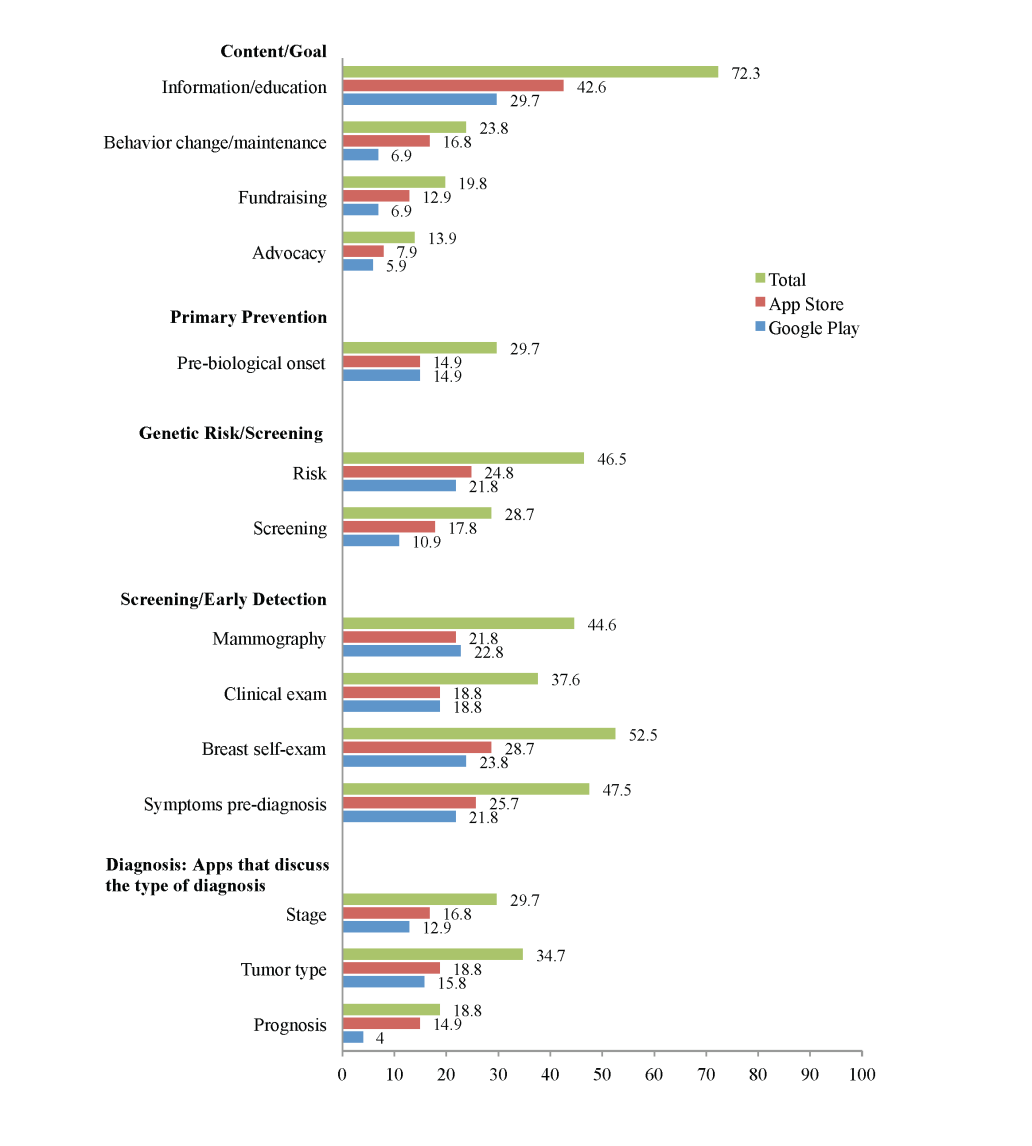
App characteristic percentages on App Store and Google Play: content/goal, primary prevention genetic risk/screening, screening/early detection, and diagnosis (N=101).

**Figure 3:**
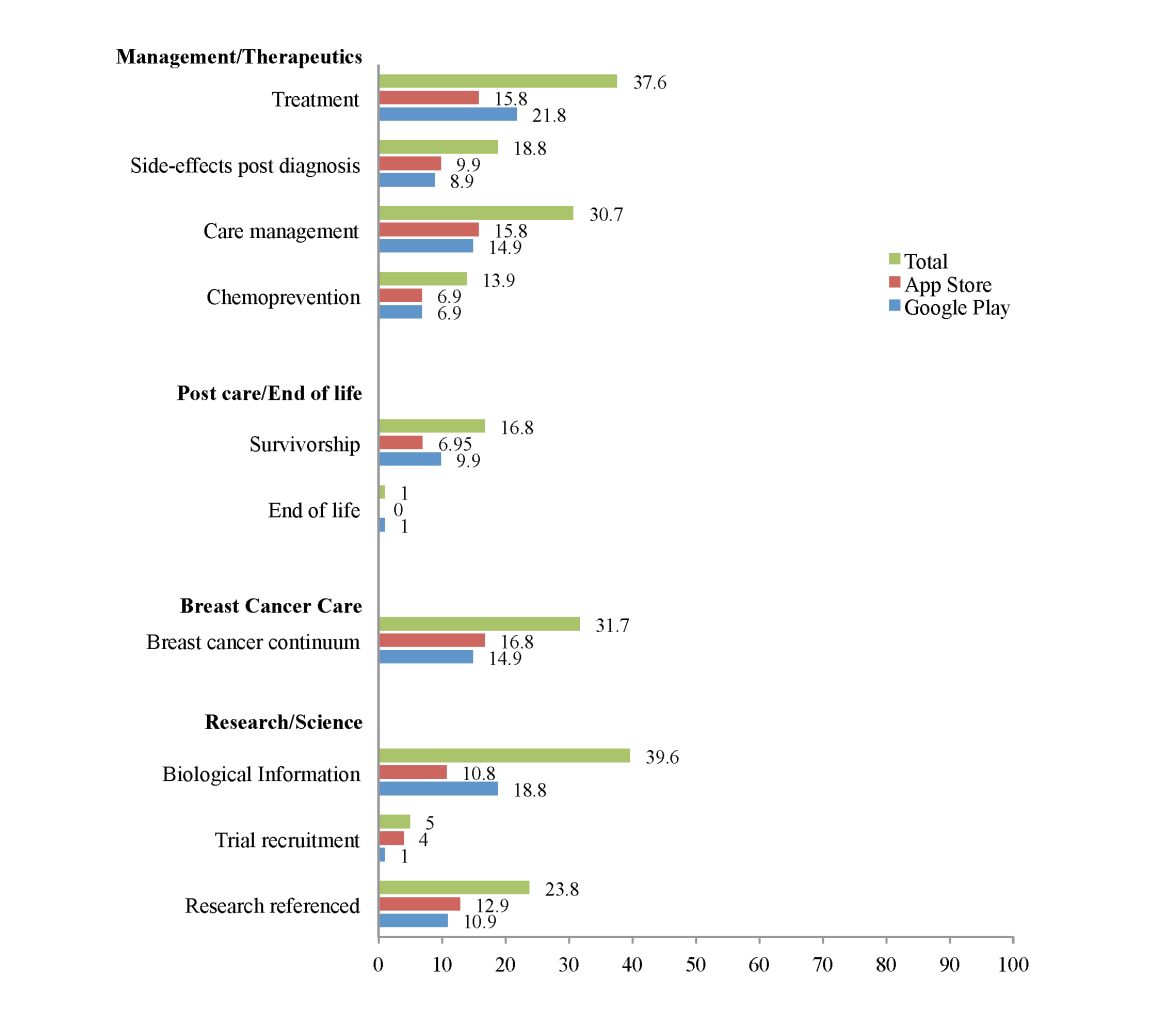
App characteristic percentages on App Store and Google Play: management/therapeutics, postcare/end of life, breast cancer care, and research/science (N=101).

**Figure 4:**
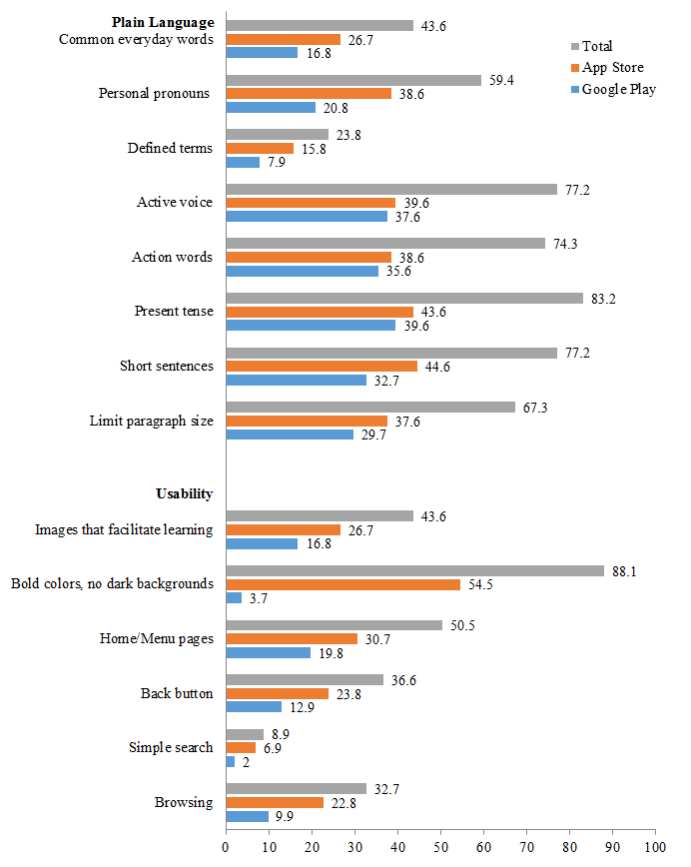
App characteristic percentages on App Store and Google Play: plain language and usability (N=101).

**Figure 5:**
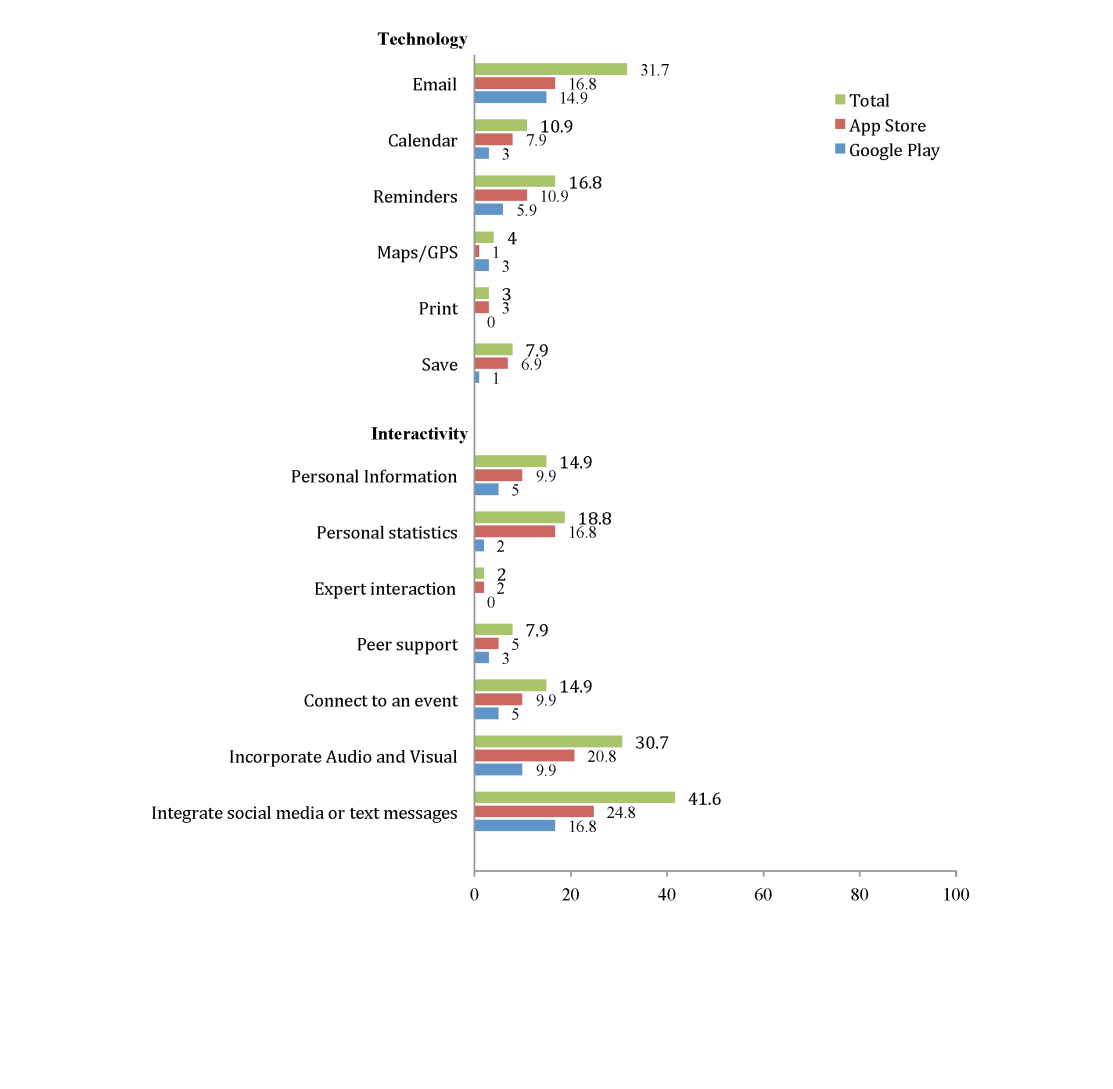
App characteristic percentages on App Store and Google Play: technology, interactivity, and approach/theoretical underpinning (N=101).

The final sample of apps that met our selection criteria and was used in the final analysis (N=101) included 44 apps (43.7%) that were available on Google Play only, 28 (27.7%) apps that were available exclusively on the App Store, and 19 (18.8%) that were available on both platforms (see Table 2 and Figures 2-5). Most apps were free (85/101, 84.2%). Of the 16 (15.8%) paid apps, prices ranged from US $0.99 to US $4.99 with a mean of US $2.15. User ratings were provided for 49 apps; the median number of ratings per app was 6 (mean 25.16, SD 74.50), and the median star rating was 4.5 of 5 (mean 4.00, SD 1.27). In total, 40 of 101 apps (39.6%) were either rated for all ages or for ages 4 years or older. Another 19 (18.8%) were rated for 12 years and older, and 17 (16.8%) were rated for 17 years and older. The categories in which the apps were placed most often were health and fitness (24.8%, 25/101) and medical (31.7%, 32/101).

**Table 2:** Characteristics of breast cancer-related apps on the App Store and Google Play (N=101).

App characteristics			n (%)
**Type of apps**			
	Android		44 (43.6)
	Apple		38 (37.6)
	Android & Apple		19 (18.8)
	Free apps		85 (84.1)
	Paid apps		16 (14.8)
**Purpose**			
	Information/education		73 (72.3)
	Behavior change/maintenance		24 (23.8)
	Fundraising		20 (19.8)
	Advocacy		14 (13.9)
**Cancer continuum**			
	**Primary prevention**		
		Prebiological onset	30 (29.7)
		Genetic risk/screening	
		Risk	47 (46.5)
		Screening	29 (28.7)
	**Evidence-based screening**		
		Mammography	45 (44.6)
		Clinical exam	38 (37.6)
	**Non-evidence-based screening**		
		Breast self-exam	53 (52.5)
		Symptoms prediagnosis	48 (47.5)
	**Diagnosis**		
		Stage	30 (29.7)
		Tumor type	35 (34.7)
		Prognosis	19 (18.8)
	**Management/Therapeutics**		
		Treatment	38 (37.6)
		Side effects	19 (18.8)
		Care management	31 (30.7)
	Chemotherapy prevention		14 (13.9)
	Survivorship		17 (16.8)
	End of life		1 (1.0)
**Research/Science**			
	Biological information		40 (39.6)
	Trial recruitment		5 (5.0)
	Research referenced		24 (23.8)
**Literate principles adherence**			
	**Plain language**		
		Common everyday words	44 (43.6)
		Personal pronouns	60 (59.4)
		Defined terms	24 (23.8)
		Active voice	78 (77.2)
		Action words	75 (74.3)
		Present tense	84 (83.2)
		Short sentences	78 (77.2)
		Limit paragraph size	68 (67.3)
	**Usability**		
		Images that facilitate learning	44 (43.6)
		Bold colors, no dark backgrounds	89 (88.1)
		Home/Menu pages	51 (50.5)
		Back button	37 (36.6)
		Simple search	9 (8.9)
		Browsing	33 (32.7)
**Technology**			
	Email		32 (31.7)
	Calendar		11 (10.9)
	Reminders		17 (16.8)
	Maps/GPS		4 (4.0)
	Print		3 (3.0)
	Save		8 (7.9)
**Interactivity**			
	Personal information		15 (14.9)
	Personal statistics		19 (18.8)
	Expert interaction		2 (2.0)
	Peer support		8 (7.9)
	Connect to an event		15 (14.9)
	Incorporate Audio and Visual		31 (30.7)
	Integrate social media or text messages		42 (41.6)

### Purpose of the Apps

The analysis included the classification of the apps’ goals by four main categories, according to the messages they advanced: (1) aimed at providing information and education about breast cancer, (2) targeted behavior change related to breast cancer, (3) included messages about fundraising, and (4) aimed at breast cancer advocacy. Most of the apps contained information/education messages (73/101, 72.3%), approximately one-quarter (24/101, 23.9%) targeted behavior change, one-fifth (20/101, 19.8%) aimed at fundraising, and a one-sixth of the apps (14/101, 13.9%) were related to advocacy. More than half of the apps focused on only one of these categories (56/101, 55.4%), 26 (25.7%) on two categories, five (5%) included three categories, and two apps included all categories. Most apps that targeted behavior change also included informational/educational goals (18/101, 75%).

### Breast Cancer Continuum

#### Primary Prevention

Almost one-third of the apps (30/101, 29.7%) presented information about primary prevention of breast cancer, such as information about diet and exercise.

#### Screening

Less than half of the apps in our sample (46/101, 45.5%) presented information about evidence-based methods of breast cancer screening (mammography and breast clinical exam). Of this subsample, 37 apps (80.4%) included information about both mammography and clinical breast exam. Eight apps contained information about breast mammography alone (17.39%), and only one contained information about clinical breast exams (2.17%).

In all, 53 apps (52.5%) discussed breast self-exam. Additional prediagnosis variables included symptoms of cancer prediagnosis, such as a lump (48/101, 47.5%) and genetic risk for breast cancer (47/101, 46.5%).

#### Diagnosis

Information about breast cancer diagnosis was included in 42 apps (41.6%). Of these apps, 15 (36%) provided information about stages of breast cancer, prognosis, and types of breast cancer tumor together; 10 apps (24%) discussed stages of breast cancer and types of breast cancer tumors together; and two (5%) provided information about stages of cancer as well as prognosis. In addition, 11 (27%) provided information about types of breast cancer tumors only, three (7%) communicated information about stages of breast cancer only, and two (5%) addressed prognosis only.

#### Treatment

Of the 101 apps, 43 (42.6%) discussed various treatment options for breast cancer patients. Of these, 18 (17.8%) provided concurrent information on (1) different treatment options, (2) possible side effects of treatment, and (3) care management of breast cancer; eight (8%) discussed treatment options and side effects; and one (2%) provided information on different treatment options and care management of breast cancer together. Eleven (26%) apps provided information about different treatment options only, and five (12%) discussed care management only.

#### Survivorship and End of Life

Seventeen of 101 apps (16.8%) discussed issues related to survivorship, such as care coordination after completion of therapeutic treatment for cancer, financial burden of cancer, late and long-term effects of breast cancer diagnosis and treatment, or health promotion after a breast cancer diagnosis. Only one of 101 apps (1.0%) discussed end-of-life hospice.

#### Research and Scientific-Related Information

In all, 40 of 101 apps (39.6%) provided biological information about breast cancer, such as the mechanism of tumor development in the breast. Only 24 apps (23.8%) cited scientific research or evidence-based guidelines to support their information. Five apps (5.0%) discussed clinical trials.

### Adherence to IOM Literate Design Principles

#### Plain Language

The median of the composite health literacy score was 5 (mean 5.06, SD 2.00), and only 13 (13%) apps had a composite plain language score of 8 of 8. A majority of the apps used present tense (84/101, 83.2%), active voice (78/101, 77.2%), short sentences (78/101, 77.2%), action words (75/101, 74.3%), short paragraph size (68/101, 67.3%), and personal pronouns such as “you” (60/101, 59.4%). However, fewer than half of the apps primarily used common and easy-to-understand words (44/101, 43.6%) and only 24 (23.8%) defined terms.

#### Usability

The median composite usability composite score was 3 (mean 2.60, SD 1.20). None of the apps contained all six usability items recommended by the IOM. Five apps (5.0%) had a composite usability score of 5 of 6. The most common usability feature was the use of bold colors without dark backgrounds (89/101, 88.1%). In all, 51 (50.5%) apps provided easy access to home/menu pages, 44 (43.6%) used images that facilitated learning, 37 (36.6%) had a back button, 33 (32.7%) were easy to browse, and nine (8.9%) had a simple search option available.

#### Graphics and Technological Features

The most common technological feature was the ability to share content via email through the app (32/101, 31.7%). In addition, eight (7.9%) apps had the option to save documents. Four apps (4.0%) connected users to maps or GPS and three (3.0%) provided users the option to print directly from the apps.

#### Interactivity

Most apps (93/101, 92.1%) did not allow the user to customize information (ie, input weight, height, and other personal measures); only eight (7.9%) offered peer support and two (2.0%) provided an “ask the expert” option.

### Adherence to IOM Plain Language and Usability Guidelines by Content Area and Goals

There was no statistically significant difference in the composite plain language scores of apps that focused on information/education content (mean 5.10, SD 1.89) and apps that did not (mean 4.96, SD 2.41; *t*_40.34_=0.26, *P=*.80). Similarly, although the composite usability scores of apps containing information/education messages (mean 2.71, SD 1.12) were somewhat higher than other apps (mean 2.32, SD 1.25), these differences were not statistically significant (*t*_99_=1.52, *P*=.13) (see Table 3).

**Table 3:** Adherence of apps to literate principles (N=101).

Content		Composite plain language score			Composite usability score		
		Mean (SD)	*t* (*df*)	*P*	Mean (SD)	*t* (*df*)	*P*
**Information/education content**			–0.26 (40.34)	.13		–0.26 (99)	.77
	No information/ education content	4.96 (2.41)			2.32 (1.25)		
	With information/education content	5.10 (1.89)			2.71 (1.12)		
**Behavior change content**			–2.80 (99)	.006		–0.30 (99)	.77
	No behavior change content	4.75 (2.00)			2.58 (1.21)		
	With behavior change content	6.04 (1.85)			2.67 (1.05)		
**Fundraising content**			–3.47 (99)	.001		0.23 (99)	.82
	No fundraising content	4.73 (1.97)			2.62 (1.11)		
	With fundraising content	6.40 (1.73)			2.55 (1.39)		
**Advocacy content**			–2.19 (99)	.031		0.11 (99)	.91
	No advocacy content	4.89 (2.07)			2.61 (1.20)		
	With advocacy content	6.14 (1.41)			2.57 (0.94)		
**Apps**			0.80 (99)	.43		–2.73 (99)	.007
	Free	5.13 (2.09)			2.47 (1.17)		
	Paid	4.69 (1.70)			3.31 (0.87)		

Apps aimed at behavior change scored higher on use of plain language (mean 6.04, SD 1.85) than apps that did not aim at behavior change (mean 0.75, SD 2.00) and the relationship was statistically significant (*t*_99_=2.80, *P*=.006). Although these type of apps also ranked slightly higher in usability (mean 2.67, SD 1.05) than the apps that did not include behavior change messages (mean 2.58, SD 1.21), the difference was not significant (*t*_99_=0.30, *P=*.77) (see Table 3).

The *t* test analysis also revealed that apps with a fundraising purpose had a higher composite plain language score (mean 6.40, SD 1.73) than those without a fundraising purpose (mean 4.73, SD 1.97) and the relationship was statistically significant (*t*_99_=3.47, *P*=.001). There was no significant difference in usability between apps that targeted fundraising (mean 2.55, SD 1.39) and those that did not (mean 2.62, SD 1.11; *t*_99_=–0.23, *P=*.82) (see Table 3).

Apps that included advocacy for breast cancer causes scored higher for plain language (mean 6.14, SD 1.41) compared to those that did not advocate for breast cancer causes (mean 4.89, SD 2.07) and the relationship was statistically significant (*t*_99_=2.19, *P*=.03). In contrast, advocacy-related apps (mean 2.57, SD 0.94) and apps that did not include advocacy (mean 2.61, SD 1.20) did not differ in their usability (*t*_99_=–1.12, *P=*.91) (see Table 3).

### Differences Between Free and Paid Apps in Adherence to IOM Guidelines

Of the 73 apps that had information/education content, 58 (79%) were free and 15 (21%) were paid. Most of the 24 apps that targeted behavior change were free (21/24, 88%), and three (13%) were paid. All 20 (100%) of the apps that aimed at fundraising were free. Of the 14 apps that that included breast cancer advocacy, 12 (86%) were free, and two (14%) were paid (see Table 3).

Free apps (mean 5.13, SD 2.09) did not differ significantly from paid apps in use of plain language (mean 4.69, SD 1.70; *t*_99_=–0.80, *P=*.43). In contrast, paid apps scored higher on usability (mean 3.31, SD 0.87) than free apps (mean 2.47, SD 1.17). The difference was statistically significant (*t*_99_=2.73, *P*=.007) (see Table 3).

### IOM Guidelines and User Ratings

Pearson correlations and *t* tests were used to examine the relationship between how well apps followed the IOM guidelines and how highly users rated those apps. Approximately half (49/101) of the apps in the sample were rated by users. There was a significant positive correlation between apps’ user ratings and their composite scores on the health literacy scale (*r*=.33, *P*=.02).

Average ratings were significantly higher for apps that used action words (mean 4.26, SD 1.07) than for apps that did not (mean 3.42, SD 1.52; *t*_47_=–2.20, *P*=.03). Ratings were also higher for apps that used the present tense (mean 4.27, SD 1.03) than for those that did not (mean 3.08, SD 1.63; *t*_12.39_=–2.28, *P*=.04).

There was no significant correlation between an app’s user rating and its composite usability score (*r*=.02, *P*=.85).

## Discussion

### Principal Findings

This study examined the availability of breast cancer-related apps, their purpose, cancer continuum-related content, adherence to literate principles design, price, and user ratings. At the time of data collection, 101 apps focusing on breast cancer were available to the public. The majority of these apps were available on Android. The proportion of Apple-only apps in the sample represented their respective share of the cellular market of 28% at the time of data collection [49], albeit not the increased likelihood of iPhone users to download apps [50]. This distribution of apps documented in this study demonstrates increasing efforts from developers to provide apps for both Android and iPhone platforms.

Although apps often have multiple purposes, the majority are designed to provide information and education. Consistent with past studies [14], these findings reveal the limited potential of the current apps available to advance breast cancer-related behavior change. Research has shown that information is important yet insufficient in changing multifaceted health behaviors [51]. The high number of apps that included information/educational content without clear guidelines for behavior change suggests limited utility of currently available apps in behavior change, despite the relative advantage of mHealth in providing interactive features that can support such change. Moreover, the content of most apps does not support evidence-based, comprehensive breast cancer-related behavior change in specific areas. These findings align with past research on cancer apps that identified few evidence-based preventive messages [14]. For instance, although research and consequently clinical guidelines in North America in the past decade concluded that breast self-examination is an ineffective and often harmful screening strategy [52,53], this strategy was featured in more apps compared to the evidence-based strategies of mammography and clinical breast exams. Involving medical professionals in design of apps [22] may improve the quality of the information they provide.

The findings also indicate that provision of information in support of treatment-related decision making emerges as an area of need. Less than half of the apps that provided information on treatment included information about possible side effects and of treatment options. This deficiency might be explained by the reluctance of developers to include medical information due to paucity of clinical expertise involvement in development of mHealth [50]. However, extant literature documented the importance of such information for women with breast cancer [54], including availability of relatively easy-to-use decision tools [55].

The analysis of the content of the apps on the cancer continuum reveals that, in contrast to primary breast cancer prevention, screening, and treatment, only a few apps focused on survivorship and only one included information about hospice care. No apps covered other aspects of end-of-life decisions and care. It is possible that information and support on end-of-life decisions and care are available on apps that are not breast cancer-specific (and consequently in apps that were not included in this sample), past research on cancer-related apps did not document such focus [56]. Therefore, these findings lend support to the need for apps that would provide evidence-based information and support behavior change and decision making following breast cancer diagnosis, with extreme need for apps on end-of-life decisions and care.

The analysis further underscores that greater adherence to literate design strategies continues to be a pressing need in breast cancer app development. Adherence to most usability design standards was low. It is possible that this low adherence relates to lack of experience or training of developers working on this relatively new platform. For instance, the literacy design principle that was most closely adhered to included use of colors, which is consistent with design of websites. In contrast, features that are arguably more significant in-app design, such as an easy browse and use of images, were not frequently included in the apps.

Similarly, the findings underscore the importance of greater attention to using plain language principles in the design of breast cancer apps. Notably, of the plain language characteristics, text level was still too high in the vast majority of apps, which demonstrates that mHealth developers, like developers of print information [57] and of Web-based information [58], are not effective in bridging the literacy divide using principles such as defining concepts. Therefore, it is evident that the need for more appropriate plain language materials persists. However, behavioral change, advocacy, and fundraising apps demonstrated higher adherence to plain language principles. It is possible that these differences indicate greater degree of professionalism of these apps’ developers.

Consistent with past studies that examined diabetes-related apps [44], paid apps were more likely than free apps to adhere to literacy guidelines. In this case, paid apps featured usability principles more frequently than free apps. As the vast majority of mobile phone users are reluctant to pay for apps [59], this finding also points at the potential for persistence of disparities between users who are able to use paid apps and those who are restricted to using only free apps. In addition, this study provides additional support to the potential of using reviewer ratings to learn about user experiences. In past research, user ratings were correlated with professional quality ratings of apps [47], but in this study they were correlated with apps’ adherence to plain language principles, thus lending further empirical support to the importance of plain language. To our knowledge, this is the first study to document such an association. In contrast, user ratings were not related to usability. Future studies should explore users’ expectations from apps’ usability in the context of breast cancer.

This study contributes to research on the use of mHealth to advance breast cancer-related education and behavior change in a few ways. First, this is the first study to focus solely on breast cancer apps. In view of the unique information, education, treatment, and support needs before and following breast cancer diagnosis [60], this focus can advance understanding on the degree to which these apps have the potential to meet these needs. In addition, past studies that examined cancer apps were limited to analyzing only iPhone apps [15], apps’ descriptions in the App Store [14], or reviews of the literature reporting on cancer-related mHealth interventions [16]. By analyzing relevant, working, uploaded apps available on both Android and iPhone platforms, this study provides a more comprehensive analysis of availability to consumers. Moreover, past studies did not examine design of cancer-related apps, including adherence to literacy design principles, and did not focus on breast cancer, whereas this study provides important insights on implementation of literate design strategies.

### Limitations

As in any research project, the methodologies utilized in this study hold inherent limitations. Specifically, systematic content analyses are helpful in providing an overview on the content available to users and its adherence to scholarly and professional standards, but are limited in their ability to shed light on users’ experiences. Moreover, previous researchers noted that content analysis of mHealth cannot link user information to app use [46]. In addition, information about the sources of the apps was not available; therefore, analysis of the relationship between app-related factors such as release dates, content source, organizational affiliation, or country of origin was not possible. Similarly, because we included only apps that focused on cancer, we did not examine other apps that might be used for cancer prevention purposes or that people might use to manage symptoms after diagnosis with cancer or survivorship and end of life. Finally, this study was conducted in the United States and, therefore, does not demonstrate availability of breast cancer-related apps in other markets or in languages other than English.

### Conclusions

Despite exciting potential for consumer engagement along the cancer continuum, availability of evidence-based breast cancer information and integration of literate design strategies to mHealth users is limited. This current state reveals that mHealth has not met its potential in engaging consumers with evidence-based information and design necessary to reduce preventable breast cancer burden and its associated disparities in health outcomes. Specifically, breast cancer-specific apps represent a limited spectrum on the cancer continuum. Therefore, this study is important in supporting the need for better-designed breast cancer apps that would adhere to evidence-based as well as to plain language and usability standards, with an extreme need for apps that focus on information necessary for medical decision making, most notably side effects, survivorship, and end of life.

As a systematic review, the goal of this study was to provide an overview of availability of breast cancer apps and their adherence to evidence-based content and design principles. Such systematic analyses are time consuming and cannot be performed by users. Further, the characteristics of such users at this point are unknown and are likely very diverse, including cancer-free individuals, cancer patients, and cancer survivors, because different apps target women at different stages on the cancer continuum. Future studies should apply additional, user-centered research methods, including surveys and community-based studies to learn about users’ experiences using apps along the breast cancer continuum.

## Abbreviations

IOM: Institute of Medicine

## References

[1] Ferlay J, Soerjomataram I, Dikshit R, Eser S, Mathers C, Rebelo M, Parkin DM, Forman D, Bray F (2015). Cancer incidence and mortality worldwide: sources, methods and major patterns in GLOBOCAN 2012. Int J Cancer.

[2] Coughlin SS, Ekwueme DU (2009). Breast cancer as a global health concern. Cancer Epidemiol.

[3] Benitez G, Desai J, Schroeder E, Nichols G, Segal J, Karter A, Steiner J, Newton K, Morales L, Pathak R, O'Connor P (2014). PS1-40: Preventable major cardiovascular events due to uncontrolled glucose, blood pressure, and lipids or active smoking in adults with diabetes with and without cardiovascular disease. Clinical Medicine & Research.

[4] Liu P, Wang J, Keating NL (2013). Expected years of life lost for six potentially preventable cancers in the United States. Prev Med.

[5] Bray F, McCarron P, Parkin DM (2004). The changing global patterns of female breast cancer incidence and mortality. Breast Cancer Res.

[6] López-Gómez M, Malmierca E, de Górgolas M, Casado E (2013). Cancer in developing countries: the next most preventable pandemic. The global problem of cancer. Crit Rev Oncol Hematol.

[7] Youlden D, Cramb SM, Dunn NA, Muller JM, Pyke CM, Baade PD (2012). The descriptive epidemiology of female breast cancer: an international comparison of screening, incidence, survival and mortality. Cancer Epidemiol.

[8] Rainie L, Poushter J (2014). Pew Research Center.

[9] Lopez MH, Gonzalez-Barrera A, Patten E (2013). Pew Research Center.

[10] Mechael PN (2009). The case for mHealth in developing countries. innovations.

[11] Boulos MN, Wheeler S, Tavares C, Jones R (2011). How smartphones are changing the face of mobile and participatory healthcare: an overview, with example from eCAALYX. Biomed Eng Online.

[12] (2011). mHealth: New Horizons for Health through Mobile Technologies: Second Global Survey on eHealth.

[13] Duggan M (2013). Pew Research Center.

[14] Bender JL, Yue RY, To MJ, Deacken L, Jadad AR (2013). A lot of action, but not in the right direction: systematic review and content analysis of smartphone applications for the prevention, detection, and management of cancer. J Med Internet Res.

[15] Pandey A, Hasan S, Dubey D, Sarangi S (2013). Smartphone apps as a source of cancer information: changing trends in health information-seeking behavior. J Cancer Educ.

[16] Davis SW, Oakley-Girvan I (2015). mHealth education applications along the cancer continuum. J Cancer Educ.

[17] Eskandar H, Land MA, Pujari S (2015). Cancer Control.

[18] Schnall R, Mosley JP, Iribarren SJ, Bakken S, Carballo-Diéguez A, Brown III W (2015). Comparison of a user-centered design, self-management app to existing mHealth apps for persons living with HIV. JMIR Mhealth Uhealth.

[19] Zhang C, Zhang X, Halstead-Nussloch R (2014). Faculty Publications.

[20] Bailey SC, Belter LT, Pandit AU, Carpenter DM, Carlos E, Wolf MS (2014). The availability, functionality, and quality of mobile applications supporting medication self-management. J Am Med Inform Assoc.

[21] Derbyshire E, Dancey D (2013). Smartphone medical applications for women's health: what is the evidence-base and feedback?. Int J Telemed Appl.

[22] Mobasheri MH, Johnston M, King D, Leff D, Thiruchelvam P, Darzi A (2014). Smartphone breast applications - what's the evidence?. Breast.

[23] Ferrero N, Morrell DS, Burkhart CN (2013). Skin scan: a demonstration of the need for FDA regulation of medical apps on iPhone. J Am Acad Dermatol.

[24] Broderick J, Devine T, Langhans E, Lemerise AJ, Lier S, Harris L (2014). National Academy of Medicine.

[25] Free C (2013). The effectiveness of mobile-health technology-based health behaviour change or disease management interventions for health care consumers: a systematic review. PLoS med.

[26] Spradley JP, McCurdy DW (1980). Anthropology: The Cultural Perspective.

[27] Spradley JP (2016). Participant Observation.

[28] Astin JA (1998). Why patients use alternative medicine: results of a national study. JAMA.

[29] Heaton J (2004). Reworking Qualitative Data.

[30] Fu MR, Axelrod D, Guth AA, Rampertaap K, El-Shammaa N, Hiotis K, Scagliola J, Yu G, Wang Y (2016). mHealth self-care interventions: managing symptoms following breast cancer treatment. Mhealth.

[31] Mackert M, Mabry-Flynn A, Champlin S, Donovan EE, Pounders K (2016). Health literacy and health information technology adoption: the potential for a new digital divide. J Med Internet Res.

[32] Demark-Wahnefried W, Pinto BM, Gritz ER (2006). Promoting health and physical function among cancer survivors: potential for prevention and questions that remain. J Clin Oncol.

[33] McCarroll ML, Armbruster S, Pohle-Krauza RJ, Lyzen AM, Min S, Nash DW, Roulette GD, Andrews SJ, von Gruenigen Vivian E (2015). Feasibility of a lifestyle intervention for overweight/obese endometrial and breast cancer survivors using an interactive mobile application. Gynecol Oncol.

[34] Goode A, Lawler SP, Brakenridge CL, Reeves MM, Eakin EG (2015). Telephone, print, and Web-based interventions for physical activity, diet, and weight control among cancer survivors: a systematic review. J Cancer Surviv.

[35] Lyons E, Baranowski T, Basen-Engquist KM, Lewis ZH, Swartz MC, Jennings K, Volpi E (2016). Testing the effects of narrative and play on physical activity among breast cancer survivors using mobile apps: study protocol for a randomized controlled trial. BMC Cancer.

[36] Hargittai E (2002). First Monday.

[37] Norman C, Skinner Harvey A (2006). eHealth literacy: essential skills for consumer health in a networked world. J Med Internet Res.

[38] Peerson A, Saunders M (2009). Health literacy revisited: what do we mean and why does it matter?. Health Promot Int.

[39] Davis T, Williams MV, Marin E, Parker RM, Glass J (2002). Health literacy and cancer communication. CA Cancer J Clin.

[40] Neter E, Brainin Esther (2012). eHealth literacy: extending the digital divide to the realm of health information. J Med Internet Res.

[41] Rios G (2013). eHealth literacy and older adults. Top Geriatr Rehabil.

[42] Ginossar T (2016). Predictors of online cancer prevention information seeking among patients and caregivers across the digital divide: a cross-sectional, correlational study. JMIR Cancer.

[43] Ginossar T (2014). Disparities and antecedents to cancer prevention information seeking among cancer patients and caregivers attending a Minority-Serving Cancer Center. J Commun Healthcare.

[44] Caburnay CA, Graff K, Harris JK, McQueen A, Smith M, Fairchild M, Kreuter MW (2014). Evaluating diabetes mobile applications for health literate designs and functionality. Prevent Chron Dis.

[45] Holzemer WL, Human S, Arudo J, Rosa ME, Hamilton MJ, Corless I, Robinson L, Nicholas PK, Wantland DJ, Moezzi S, Willard S, Kirksey K, Portillo C, Sefcik E, Rivero-Méndez M, Maryland M (2009). Exploring HIV stigma and quality of life for persons living with HIV infection. J Assoc Nurses AIDS Care.

[46] Molina Y, Ramirez-Valles J (2013). HIV/AIDS stigma: measurement and relationships to psycho-behavioral factors in Latino gay/bisexual men and transgender women. AIDS Care.

[47] Stoyanov S, Hides L, Kavanagh DJ, Zelenko O, Tjondronegoro D, Mani M (2015). Mobile app rating scale: a new tool for assessing the quality of health mobile apps. JMIR Mhealth Uhealth.

[48] Abroms L, Padmanabhan N, Thaweethai L, Phillips T (2011). iPhone apps for smoking cessation: a content analysis. Am J Prev Med.

[49] Gant LM, Nagda BA, Brabson HV, Jayaratne S, Chess WA, Singh A (1993). Effects of social support and undermining on African American workers' perceptions of coworker and supervisor relationships and psychological well-being. Soc Work.

[50] Santen SA, Deiorio NM, Gruppen LD (2012). Medical education research in the context of translational science. Acad Emerg Med.

[51] Ng T, F DC (2012). Age and innovation-related behavior: the joint moderating effects of supervisor undermining and proactive personality. J Organiz Behav.

[52] Wohl AR, Galvan FH, Carlos J, Myers HF, Garland W, Witt MD, Cadden J, Operskalski E, Jordan W, George S (2013). A comparison of MSM stigma, HIV stigma and depression in HIV-positive Latino and African American men who have sex with men (MSM). AIDS Behav.

[53] Freeman E (2016). Understanding HIV-related stigma in older age in rural Malawi. Soc Sci Med.

[54] Elwyn G, Lloyd A, Joseph-Williams N, Cording E, Thomson R, Durand M, Edwards A (2013). Option grids: shared decision making made easier. Patient Educ Couns.

[55] Charles C, Gafni A, Whelan T (1999). Decision-making in the physician-patient encounter: revisiting the shared treatment decision-making model. Soc Sci Med.

[56] LaBresh K, Ariza AJ, Lazorick S, Furberg RD, Whetstone L, Hobbs C, de JJ, Salinas IG, Bender RH, Binns HJ (2014). Adoption of cardiovascular risk reduction guidelines: a cluster-randomized trial. Pediatrics.

[57] McGaghie W (2010). Medical education research as translational science. Sci Transl Med.

[58] Price D, Wagner DP, Krane NK, Rougas SC, Lowitt NR, Offodile RS, Easdown LJ, Andrews MA, Kodner CM, Lypson M, Barnes BE (2015). What are the implications of implementation science for medical education?. Med Educ Online.

[59] Zhong N (2013). Google play is not a long tail market: an empirical analysis of app adoption on the Google play app market. Proceedings of the 28th Annual ACM Symposium on Applied Computing.

[60] Ashing-Giwa K, Padilla G, Tejero J, Kraemer J, Wright K, Coscarelli A, Clayton S, Williams I, Hills D (2004). Understanding the breast cancer experience of women: a qualitative study of African American, Asian American, Latina and Caucasian cancer survivors. Psychooncology.

